# Comment on Sallam, M. ChatGPT Utility in Healthcare Education, Research, and Practice: Systematic Review on the Promising Perspectives and Valid Concerns. *Healthcare* 2023, *11*, 887

**DOI:** 10.3390/healthcare11212819

**Published:** 2023-10-25

**Authors:** Emilio Moreno, Luis Adrian Alvarez-Lozada, Francisco Javier Arrambide-Garza, Alejandro Quiroga-Garza, Rodrigo Enrique Elizondo-Omaña

**Affiliations:** Human Anatomy Department, School of Medicine, Universidad Autonoma de Nuevo Leon, Monterrey 64460, Mexico; emilio.moreno@uanl.edu.mx (E.M.); doctor.luisadrian@gmail.com (L.A.A.-L.); arrambidefrancisco@gmail.com (F.J.A.-G.); rod_omana@yahoo.com (R.E.E.-O.)

We read with great interest and applaud the recently published review paper regarding ChatGPT and its implications in research and education [1]. This is one of the first papers to synthesize the possible benefits and potential limitations of this Artificial Intelligence (AI)-based conversational large language model (ChatGPT). This will be an important reference in future studies of this topic, as AI continues to play a larger role in academic and scientific work, with ethical concerns arising and the risk of misinformation [2]. 

After reading this review, we have several concerns. As long as ChatGPT and other AI-based platforms exist, their use will continue to increase. Guidelines and recommendations must be generated to motivate users to properly and purposefully integrate ChatGPT into their work as a tool to make it more efficient and with higher quality but with rigorous supervision and human input. The conclusions drawn from this article should be approached with caution due to its limitations. Although it is a systematic review, it lacks some key components of the PRISMA Checklist [3]. It is recommended that all systematic reviews be previously registered in the International Prospective Register of Systematic Reviews (PROSPERO). The search strategy (which is not made available) was performed in only two databases, limiting the reach of the available work. The record selection process should be performed in duplicate with the chance-adjusted inter-rater agreement estimated using the Kappa statistic to reduce the risk of bias in the selection process [4]. Due to the qualitative nature of the research question, the selection of articles may have exhibited the author’s specific viewpoint. A methodological quality assessment should be performed with a validated scale according to the study design and included in the results so readers may understand the risk of bias in the included studies [4]. Qualitative analysis was performed; however, the method used was not specified. It is also unclear whether the categorization of the results was undertaken a priori or after the results were obtained due to the absence of a PROSPERO registration.

Our particular point of view is that ChatGPT is a tool that students and medical staff are going to be using for a long time and should be considered by researchers and educators. This is why the publication of this article is valuable for the scientific community to start understanding and, more importantly, to start accepting the fact that the use of AI is inevitable. One of the main take-away points that could be obtained from this review is that a lot more research is needed to improve the use of this AI large language model, and special focus should be applied to teaching users the responsibility of purposefully using AI in their academic, research, and clinical activities [5].
